# Impact of Primary Spoken Language as a Social Determinant of Health on Cardiopulmonary Education and Use: Pilot Study

**DOI:** 10.5811/westjem.47910

**Published:** 2026-01-03

**Authors:** Charles LeNeave, Brian Meier, Heather Liffert, John C. Perkins

**Affiliations:** * Virginia Tech Carilion School of Medicine, Roanoke, Virgina; † Virginia Tech Carilion School of Medicine, Department of Emergency Medicine, Roanoke, Virgina; ‡ Carilion Clinic, Roanoke, Virginia; § Compress and Shock Foundation, Roanoke, Virginia

## Abstract

**Introduction:**

Over 350,000 out-of-hospital cardiac arrests occur annually in the United States, with neurologically intact survival below 10%. Recent literature demonstrates that survival is lower in communities of color and non-English speakers. Social determinants of health, such as healthcare access, language, and literacy, may serve as barriers to receiving cardiopulmonary resuscitation (CPR) education and using the skills learned. Current research is sparse on identifying barriers contributing to the lack of CPR education and use in non-English speaking communities. We hypothesized that barriers to CPR education and use differ between English- and Spanish-speaking learners. This study provides insights into how classes could be tailored to address disparities in CPR education and use.

**Methods:**

In this cross-sectional study we used survey-based research to assess the knowledge, comfort, and perceived barriers to activating the 9-1-1 system and performing bystander CPR. Participants were recruited using convenience sampling at community-based events in Roanoke, Virginia. We directly compared responses between language groups using Fisher tests within R, adjusting for various demographic factors.

**Results:**

We collected 367 surveys from the 550 participants (estimated 50 attendees each for 11 events) for a response rate of 66.7%. Of the surveys collected, 231 (63%) were in English and 136 (37%) in Spanish. Spanish-speakers were more concerned with immigration status (7% vs 1%), doing something wrong (14% vs 7%), and language barrier (31% vs 1%) compared to English-speakers when asked why they may not call 9-1-1. We found that 72% of English-speakers would have no problem calling 9-1-1, compared to only 16% of Spanish-speakers. Regardless of language, the most prevalent barrier to initiating CPR was the “fear of doing something wrong” with 49% of Spanish-speakers and 28% of English-speakers endorsing this as a barrier. Only 10% of Spanish speakers would have no concerns starting CPR, compared to 54% of English-speakers. Language was reported by 21% of Spanish-speakers vs 2% of English-speakers as a barrier to administering CPR.

**Conclusion:**

Results of this pilot study highlight that Spanish-speaking respondents were less comfortable calling 9-1-1 and initiating CPR compared to English-speaking respondents. While there were some shared barriers between the groups, Spanish-speaking respondents were more likely to identify a barrier overall. These results suggest that marginalized communities would benefit from tailored educational models that address their unique challenges. Further research is necessary to better understand how social determinants of health serve as barriers to CPR education/use in specific communities.

## INTRODUCTION

In 2022, the American Heart Association (AHA) reported 356,461 out-of-hospital cardiac arrests (OHCA) in the United States with less than 9% surviving to hospital discharge.^1^ A key piece to responding to OHCA is the “chain of survival” concept. The chain of survival is a sequence of time-sensitive events or “links”: early recognition of cardiac arrest and activation of the emergency-response system; immediate, high-quality cardiopulmonary resuscitation (CPR); rapid defibrillation; basic and advanced response of emergency medical services (EMS); and Advanced Life Support and post-arrest care. 2 Of these, CPR and early defibrillation using an automated external defibrillator (AED) are the most effective lay responder interventions.^2^ Cardiopulmonary resuscitation is considered a bridge to defibrillation and largely functions to optimize the chances for neurologically intact recovery following return of spontaneous circulation. Permanent neurologic injury and chance for survival are estimated to decrease by 10% with every minute that passes between OHCA and initiation of CPR or use of an AED.^1^,^3^

Despite this, lay responder delivery of CPR occurs in less than 40% of OHCA victims.^4^ This may be due in part to a misunderstanding or lack of knowledge of the capabilities of CPR, resuscitative measures, and other healthcare interventions.^5^ Early access and use of an AED in OHCA is considered the most likely avenue to increase OHCA survival; however, use of an AED prior to EMS arrival is only 3.7%.^6^ Research published in 2000 demonstrated the survival benefits from early use of AED in OHCA, using casinos as the study arena.^7^ Survival to discharge was 74% in arrests in ventricular fibrillation that had an AED used within three minutes.^7^

However, OHCA survival statistics can be misleading as there are discrepancies in lay rescuer CPR. Previous literature has found that globally, women are less likely to receive CPR from lay rescuers compared to men.^8^ Non-White individuals have repeatedly been reported to have lower rates of lay rescuer CPR when compared to White individuals. Disparities in Black and Hispanic populations receiving lay rescuer CPR are well documented.^9^–^11^ Being Black or Hispanic correlated to being 10–20% less likely to receive bystander CPR following a witnessed OHCA, regardless of the event occurring in public or at home.^9^ There is limited research on the link between language barriers and lay rescuer CPR, but there is evidence that underuse of 9-1-1, delays in care, and survival outcomes can be attributed to non-English speakers.^12^–^14^

These disparities are in part due to social determinants of health (SDoH) or non-medical factors that influence health outcomes.^15^ The five domains of SDoH are commonly viewed as the following: economic stability; education access/quality; healthcare access/quality; neighborhood/built environment; and social/community context.^16^ Lack of equal access to CPR education is affected by each of these factors. In studies that have examined CPR education, there are lower rates of accessing education among individuals who are women, non-White, older, and of a lower socioeconomic status.^1^,^17^, ^18^ Several factors have been identified that may represent barriers to CPR education/access, as well as activation of the chain of survival. Examples of these reported factors include language barrier, class cost, fear/distrust of law enforcement, fear over immigration status, liability concerns, and fear of causing harm to the victim.^19^–^21^

**Population Health Research Capsule**

What do we already know about this issue?

*In communities of color, healthcare access, language, and literacy may serve as barriers to receiving CPR education and using those skills. Current research is sparse*.

What was the research question?

*We investigated barriers to serving as a lay rescuer and identified associations between socioeconomic factors (primarily language) and those barriers*.

What was the major finding of the study?

*The odds of Spanish-speaking respondents being comfortable calling 9-1-1 were 87% lower compared to English-speaking individuals (OR 0.13; 95% CI, 0.06–0.25; P < .001)*.

How does this improve population health?

*Further research is needed to explore how social determinants of health and language barriers impact the out-of-hospital cardiac arrest chain of survival*.

These barriers may be seen in several groups who are limited in accessing CPR education and addressing these barriers is critical to increasing awareness and education. In this study we aimed to add to the identification of community-specific barriers. Our objectives were to 1) investigate barriers to successful activation of the chain of survival and serving as a lay rescuer, and 2) identify any associations between socioeconomic factors (primarily language) and barriers—specifically in Roanoke, Virginia. We hypothesized that barriers identified by participants who primarily spoke English would differ from those who primarily spoke Spanish.

## METHODS

This study consisted of cross-sectional, survey-based research. A survey instrument (Appendix A) was designed to assess the knowledge, comfort, and perceived barriers to activating the 9-1-1 system and performing lay rescuer CPR.

### Study Sample

Participants were recruited in person between June 2023–June 2024 using a convenience sampling strategy at community-based events in Roanoke, Virginia. These included free, non-certification, public CPR/AED classes taught in English and/or Spanish, as well as non-medical gatherings in association with community organizations. All persons ≥ 13 years of age were eligible to take the survey. Participants were asked to select from surveys in English or Spanish based on their primary spoken language. Community members who spoke a language other than Spanish or English were not administered surveys. Survey sites were selected based through convenience sampling and did not have specific selection criteria. They included English- and Spanish-speaking sites: churches; community CPR classes; local soccer games; community skills classes; and local restaurants. There were no other eligibility criteria. Surveys did not collect any private health information. Surveys were completed on paper and entered/stored via Research Electronic Data Capture (REDCap), housed at Virginia Tech Carilion School of Medicine.^22^,^23^

### Survey Design and Measures

Demographic information (ZIP code, age range, sex, race/ethnicity, education level, income) was collected via the survey instrument. Survey items related to perceived barriers to calling 9-1-1 or starting CPR, asked participants to consider the following barriers: law enforcement; immigration status; cost of CPR/AED training; fear of doing something wrong; language barrier; and concern for violence. In addition, participants had the option to mark “other” or no barrier to calling 9-1-1/starting CPR. The survey was created in English and then translated into Spanish by a fluent speaker and verified among multiple, bilingual, native Spanish-speakers to ensure retained accuracy and meaning. We excluded from the study any surveys that were collected outside the Roanoke area, as well as surveys indicating residence via ZIP code. A complete list of the included ZIP codes can be found in Appendix B. Any missing values were treated as “NA.”

### Statistical Analysis

We conducted all analyses and data management using R 4.3.1 (R Foundation for Statistical Computing, Vienna, Austria).^24^ Survey responses were directly compared between language groups (primary outcome) using Fisher tests. To analyze the association between language and perceived barriers, a model was built for each of the barriers that demonstrated statistical significance of *P* < 0.001 after the Fisher test. This choice was made to increase interpretability and to provide a baseline for more complex models in the future. As a result, each model was adjusted for age group, household income, and education level before being run against the outcome of survey language. For all models, the reference values were based upon previous literature to compare any relevant results to those of past studies. For age group, the reference was 26–40 years of age. For household income, the reference was between $10,000-$30,000. For education level, the reference was two years of college.

We ran Fisher tests using the “testExact” argument within the “CreateTableOne” function from the package “tableone.”^25^ Models were built using the “glm” function from the package “stats.”^24^ Regression coefficient estimates were exponentiated, and we calculated 95% confidence intervals. Results of the regressions are presented as odds ratios (OR) with 95% CI and *P*-values.

## RESULTS

### Survey Response

In this study survey, response was voluntary and not incentivized; hence, the rate of participation at events varied. All events contained between 10–100 participants. Of the surveys distributed in Roanoke, VA, details were not kept on the number of event attendees and how many attendees completed surveys during the initial distribution of surveys. This information was only recorded at the last three events (of 11 participants each) where surveys were distributed. As a result, we were unable to calculate the complete survey response rate. With an approximation of 50 attendees per event and 11 events, there were approximately 550 total participants. Of those 550 participants, 367 surveys were collected for a response rate of 66.7%. Upon entering an event, participants were handed a survey and a writing tool. They were asked to complete it, if they were willing to, and to hand it back to a volunteer when finished. Once surveys were completed and returned, the CPR/AED education began. We believe the overall response rate was likely similar to this as there were no major changes in the structure of the training sessions or survey-collection methods between events.

### Descriptive Statistics

A total of 367 surveys were collected: 231 in English and 136 in Spanish. Table 1 presents demographic information of survey participants.

Of English-speaking participants 72% indicated they would have no problem calling 9-1-1, compared to only 16% of Spanish-speaking participants who answered the same question (Table 2). In addition, Spanish speakers expressed higher rates of concern over the following barriers: immigration status (7%); fear of doing something wrong (14%); cost (14%); and language barrier (31%) when compared to English-speaking participants.

Regardless of language spoken, the most prevalent barrier to initiating CPR was the “fear of doing something wrong” with 49% of Spanish speaking] and 28% of English-speaking participants endorsing this as a barrier (Table 3). Only 10% of Spanish speakers stated they would have no concerns starting CPR, compared to 54% in the English-speaking group. Language barrier was indicated by 21% of Spanish speakers as a barrier to administering lay rescuer CPR, compared to 2% in the English-speaking group.

### Regressions

#### Barriers to Calling 9-1-1

Primary language, age, and education level were significantly associated with willingness to call 9-1-1 (Table 5). The odds of Spanish-speaking respondents being willing to call 9-1-1 were 87% lower compared to English-speaking individuals (OR 0.13; 95% CI, 0.06–0.25; *P* < .001). Primary language and age were significantly associated with barriers to calling 9-1-1. Spanish-speaking respondents were over 40 times more likely to indicate a language barrier to calling 9-1-1 when compared to English-speaking respondents (OR 40.08; 95% CI, 9.99–280.8; *P* < .001). Respondents between 13–25 years of age were 73% less likely to indicate language as a barrier to calling 9-1-1 compared to respondents 26–40 years of age (OR 0.27; 95% CI, 0.07–0.89; *P* = .04).

#### Barriers to Starting Cardiopulmonary Resuscitation

Primary language and age were significantly associated with willingness to start CPR (Table 6). The odds of Spanish-speaking respondents being willing to start CPR were 89% lower compared to English-speaking individuals (OR 0.11; 95% CI, 0.05–0.24; *P* < .001). Respondents 56–70 of age were 4.79 times more likely to indicate no problem starting CPR compared to those 13–25 (95% CI, 2.07–11.51; *P* < .001). Primary language and age were significantly associated with a fear of doing something wrong as barriers to starting CPR. Spanish-speaking respondents were 1.91 times more likely to indicate fear of doing something wrong as a barrier to starting CPR, compared to English-speaking respondents (95% CI,1.02, 3.61; *P* = .44).

Primary language and income were significantly associated with barriers to starting CPR.

## DISCUSSION

In this pilot study, we found language-specific barriers to successfully activating the chain of survival and serving as a lay rescuer. The chain of survival concept begins with the recognition of sudden cardiac arrest and initiation of the emergency response system through calling 9-1-1, followed by the initiation of high-quality CPR. There is limited research investigating associations between language barriers and lay rescuer CPR, but there is evidence that underuse of 9-1-1 and delays in care can be attributed to non-English speakers.^12^–^14^ This study demonstrates that Spanish-speakers expressed higher rates of concern around certain barriers, compared to English-speakers. Additionally, several sociodemographic factors were associated with certain barriers.

Nearly three-quarters of our Spanish-speaking survey respondents reported they would have some problem with calling 9-1-1, including the language barrier. Other barriers to calling 9-1-1 that the Spanish- speaking respondents reported were the following: immigration status; “fear of doing something wrong”; and cost. Although there was no significant association between immigration status and demographic variables, it is important to recognize that we collected data prior to the current administration’s increase in immigration enforcement. Regardless of language spoken, the most prevalent barrier to initiating CPR was the “fear of doing something wrong” with 49% of Spanish-speaking participants and 28% of English-speaking participants endorsing this as a barrier. Almost 90% of Spanish speakers stated they would have some problem starting CPR, compared to approximately 46% of the English speakers. Language was reported by 21% of Spanish speakers vs 2% of English speakers as a barrier to administering lay rescuer CPR. This is consistent with previous research on barriers to calling 9-1-1 and initiating CPR.^18^,^26^

Second to language the most prevalent factor in barriers to calling 9-1-1 was age. Associations were seen between age and multiple barriers, including cost and fear of doing something wrong. Respondents between 56–70 years of age had higher odds of indicating they had no problem calling 9-1-1 and lower odds of reporting cost as a barrier compared to those 26–40 years of age. In the 13–25 cohort, the odds of reporting language as a barrier to calling 9-1-1 were lower and odds of reporting fear of doing something wrong was higher compared to those 26–40 years of age. Primary language was associated with a barrier to calling and willingness to call 9-1-1. Education level did demonstrate association with willingness to call 9-1-1.

Spanish-speaking respondents were 89% less likely to have no problem starting CPR compared to English-speaking respondents. In addition, Spanish-speaking respondents were 18.24 times more likely to indicate language as a barrier to starting CPR and 1.91 times more likely to indicate fear of doing something wrong as a barrier to starting CPR, compared to English-speaking respondents. Compared to those 26–40 years of age, respondents who were 41–70 years of age were less likely to report the fear of doing something wrong as a barrier to starting CPR.

These results build on previous findings that non-English speaking individuals face language barriers to calling 9-1-1 and starting CPR.^11^–^13^ It also highlights the sociodemographic factors that have higher odds of being associated with certain barriers. This highlights the disparity in CPR education penetration equitably to all communities and the need for community-specific education.

## LIMITATIONS

This was a pilot study focused on survey research in Southwest Virginia, specifically Roanoke, and should be viewed in light of several limitations. First, the majority of the Spanish-speaking individuals in the Roanoke area are from Honduras and Mexico.^26^ Due to a variety of dialects within the Spanish language, the lack of formal testing for cultural or linguistic equivalence before translating the survey, and the lack of pretesting or cognitive interviewing to validate the survey, cross-group equivalence and generalizability may be limited. Interpretability may be limited due to race and ethnicity measurements that did not follow federal standards by combining race and ethnicity into a single item. Efforts were made to reduce sampling bias by recruiting subjects from similar environments, but it is possible both groups are not fully represented by the sample data. Sample size was determined by feasibility and available participants. Given convenience sampling without a concretely calculable response rate, the possibility of selection and non-response bias is higher.

Third, regression models were built to account for some sociodemographic factors but not all. Age group, highest level of education, and household income were controlled for in models as they demonstrated statistical significance. Although race did demonstrate statistical significance, it was not included in the model. This was to avoid over-adjustment of the model along with other social determinants of health. Prior to the study start, a priori power analysis was not run. Sample size was determined by feasibility and available participants. As a result, the study may be underpowered to detect small-to-moderate differences across language groups and subgroups, increasing the risk of type II error and contributing to wide confidence intervals.

Given that English and Spanish were the only two languages studied, the barriers identified and their impact may vary among groups that speak other languages. Additionally, the role of religion or other cultural factors not investigated may play crucial roles in affecting willingness to perform CPR or call 9-1-1 and were not investigated in this study. Despite survey anonymity, the possibility of social desirability bias impacting results with self-reported data is a possible factor to consider. Other sociodemographic factors were not controlled for and could be potential confounders. Finally, in conducting this pilot study, our aim was to identify areas of future research and tangible goals for improved CPR education; this study lays the groundwork for such inquiry. Finally, we did not account for all barriers that may affect the various steps in the chain of survival for Spanish-speaking individuals.

## CONCLUSION

Spanish-speaking survey respondents were significantly less likely to feel comfortable calling 9-1-1 and initiating CPR compared to those who spoke English. Further study investigating why this is the case, and what barriers are faced by other communities may yield greater ability to address the disparities in bystander CPR use and reception. This study demonstrates the need for further research exploring how social determinants of health impact the chain of survival in other non-English-speaking communities. Furthermore, this study underscores the need for community-tailored CPR education that includes information on the US emergency response system that is language-accessible and culturally appropriate.

## Supplementary Information





## Figures and Tables

**Table 1:** Sociodemographic characteristics of respondents to a survey regarding their knowledge, comfort, and perceived barriers to performing bystander CPR.

	Overall (N = 367)	English (n = 231)	Spanish (n = 136)	P-value
Age				< .001*
13–25 years	62 (17.1%)	30 (13.2%)	32 (23.7%)	
26–40 years	75 (20.7%)	25 (11.0%)	50 (37.0%)	
41–55 years	82 (22.6%)	50 (21.9%)	32 (23.7%)	
56–70 years	96 (26.4%)	77 (33.8%)	19 (14.1%)	
71–85 years	41 (11.3%)	39 (17.1%)	2 (1.5%)	
>85 years of age	7 (1.9%)	7 (3.1%)	0 (0.0%)	
Sex				.11
Female	226 (62.1%)	147 (63.9%)	79 (59.0%)	
Male	133 (36.5%)	78 (33.9%)	55 (41.0%)	
Other	5 (1.4%)	5 (2.2%)	0 (0.0%)	
Education Level				< .001*
2 years of college	69 (19.4%)	59 (26.2%)	10 (7.6%)	
4 years of college	81 (22.8%)	48 (21.3%)	33 (25.2%)	
Graduate School	57 (16.0%)	50 (22.2%)	7 (5.3%)	
High School	137 (38.5%)	65 (28.9%)	72 (55.0%)	
Primary School	12 (3.4%)	3 (1.3%)	9 (6.9%)	
Race/Ethnicity				< .001*
Asian or Pacific Islander	2 (0.8%)	2 (1.0%)	0 (0.0%)	
Black	94 (35%)	94 (48%)	0 (0.0%)	
Hispanic or Latino	68 (26%)	1 (0.5%)	67 (94%)	
Multiracial or biracial	7 (2.6%)	6 (3.1%)	1 (1.4%)	
Native American or Alaskan Native	1 (0.4%)	1 (0.5%)	0 (0.0%)	
White	93 (35%)	90 (46%)	3 (4.2%)	
Household Income				< .001*
Under $10,000	51 (15.4%)	19 (9.0%)	32 (26.2%)	
$10,000–$30,000	58 (17.5%)	25 (11.9%)	33 (27.0%)	
$30,000–$60,000	85 (25.6%)	49 (23.3%)	36 (29.5%)	
Over $60,0000	138 (41.6%)	117 (55.7%)	21 (17.2%)	
Under $10,000	51 (15.4%)	19 (9.0%)	32 (26.2%)	
“Have you received CPR/AED training prior to today?”				< .001*
Yes, both	73 (28.0%)	65 (33.5%)	8 (11.9%)	
Yes, CPR only	65 (24.9%)	56 (28.9%)	9 (13.4%)	
Yes, AED only	4 (1.5%)	4 (2.1%)	0 (0.0%)	
No	119 (45.6%)	69 (35.6%)	50 (74.6%)	

* Statistically significant estimates at P < .05.

*CPR*, cardiopulmonary resuscitation; *AED*, automated external defibrillator.

**Table 2:** Survey respondents’ reported barriers to calling 9-1-1.

	Overall (N = 367)	English (n = 231)	Spanish (n = 136)	P-value
Willingness to call 9-1-1				< .001*
No	179 (48.8%)	65 (28.1%)	114 (83.8%)	
Yes	188 (51.2%)	166 (71.9%)	22 (16.2%)	
Law enforcement				.56
No	349 (95.1%)	218 (94.4%)	131 (96.3%)	
Yes	18 (4.9%)	13 (5.6%)	5 (3.7%)	
Immigration status				.01*
No	355 (96.7%)	228 (98.7%)	127 (93.4%)	
Yes	12 (3.3%)	3 (1.3%)	9 (6.6%)	
Cost				.03*
No	333 (90.7%)	216 (93.5%)	117 (86.0%)	
Yes	34 (9.3%)	15 (6.5%)	19 (14.0%)	
Fear of doing something wrong				.03*
No	333 (90.7%)	216 (93.5%)	117 (86.0%)	
Yes	34 (9.3%)	15 (6.5%)	19 (14.0%)	
Language barrier				< .001*
No	322 (87.7%)	228 (98.7%)	94 (69.1%)	
Yes	45 (12.3%)	3 (1.3%)	42 (30.9%)	
Concern for violence				.68
No	349 (95.1%)	221 (95.7%)	128 (94.1%)	
Yes	18 (4.9%)	10 (4.3%)	8 (5.9%)	
Other				.21
No	362 (98.6%)	226 (97.8%)	136 (100.0%)	
Yes	5 (1.4%)	5 (2.2%)	0 (0.0%)	

* Statistically significant estimates at P < .05.

**Table 3:** Survey respondents’ reported barriers to starting cardiopulmonary resuscitation.

	Overall (N = 367)	English (n = 231)	Spanish (n = 136)	P-value
Willingness to call 9-1-1				< .001*
No	229 (62.4%)	107 (46.3%)	122 (89.7%)	
Yes	138 (37.6)	124 (53.7%)	14 (10.3%)	
Law enforcement				1.00
No	353 (96.2%)	222 (96.1%)	131 (96.3%)	
Yes	14 (3.8%)	9 (3.9%)	5 (3.7%)	
Immigration status				1.00
No	360 (98.1%)	227 (98.3%)	133 (97.8%)	
Yes	7 (1.9%)	4 (1.7%)	3 (2.2%)	
Cost				1.00
No	359 (97.8%)	226 (97.8%)	133 (97.8%)	
Yes	8 (2.2%)	5 (2.2%)	3 (2.2%)	
Fear of doing something wrong				< .001*
No	235 (64.0%)	166 (71.9%)	69 (50.7%)	
Yes	132 (36.0%)	65 (28.1%)	67 (49.3%)	
Language barrier				< .001*
No	335 (91.3%)	227 (98.3%)	108 (79.4%)	
Yes	32 (8.7%)	4 (1.7%)	28 (20.6%)	
Concern for violence				.73
No	359 (97.8%)	225 (97.4%)	134 (98.5%)	
Yes	8 (2.2%)	6 (2.6%)	2 (1.5%)	
Other				.56
No	358 (97.5%)	224 (97.0%)	134 (98.5%)	
Yes	9 (2.5%)	7 (3.0%)	2 (1.5%)	

* Statistically significant estimates at P < .05.

**Table 5:** Regression analysis between primary language and barriers to calling 9-1-1 among survey participants.

	Willingness to call 9-1-1		Immigration status		Cost		Fear of doing something wrong		Language Barrier	

	OR	95% CI	OR	95% CI	OR	95% CI	OR	95% CI	OR	95% CI

Primary Language										
English	REF	REF	REF	REF	REF	REF	REF	REF	REF	REF
Spanish	0.13*	0.06, 0.25	2.28	0.40, 19.44	1.45	0.59, 3.65	1.18	0.43, 3.25	40.08*	9.99, 280.8
Age										
13–25 years	0.43	0.16, 1.09	3.69	0.79, 21.6	2.02	0.74, 5.69	8.46*	2.78, 30.1	0.27*	0.07, 0.89
26–40 years	REF	REF	REF	REF	REF	REF	REF	REF	REF	REF
41–55 years	1.43	0.65, 3.19	0.73	0.09, 4.93	0.71	0.24, 2.00	0.62	0.15, 2.41	0.89	0.33, 2.34
56–70 years	4.89*	2.09, 11.9	**	**	0.17*	0.02, 0.73	0.19	0.01, 1.24	1.81	0.49, 6.86
71–85 years	3.07*	1.08, 9.37	**	**	**	**	**	**	4.1	0.39, 43.61
> 85 years of age	0.49	0.06, 3.50	4.71	0.14, 107.28	2.38	0.10, 23.94	4.85	0.19, 59.98	**	**
Education Level										
2 years of college	0.68	0.29, 1.59	**	**	2.03	0.59, 6.64	1.36	0.31, 5.24	0.72	0.16, 2.86
4 years of college	0.43*	0.19, 0.95	0.44	0.02, 3.41	1.88	0.62, 5.66	2.74	0.79, 9.88	0.8	0.27, 2.25
Graduate School	0.61	0.24, 1.54	1.71	0.07, 19.22	0.9	0.12, 4.22	0.45	0.02, 3.10	**	**
High School	REF	REF	REF	REF	REF	REF	REF	REF	REF	REF
Primary School	0.66	0.09, 3.03	1.24	0.06, 9.45	0.47	0.02, 2.86	2.62	0.46, 12.87	1.59	0.33, 7.52
Household Income										
Under $10,000	REF	REF	REF	REF	REF	REF	REF	REF	REF	REF
$10,000–$30,000	1.22	0.42, 3.59	3.57	0.73, 21.19	0.52	0.14, 1.78	1.34	0.34, 5.33	1.22	0.42, 3.59
$30,000–$60,000	2.37	0.91, 6.43	0.83	0.10, 5.85	0.47	0.14, 1.51	1	0.26, 3.78	2.37	0.91, 6.43
> $60,0000	1.81	0.68, 4.98	0.36	0.01, 3.93	0.52	0.15, 1.75	1.01	0.27, 3.85	1.81	0.68, 4.98

* Statistically significant estimates at P < .05.

** Estimate unable to be obtained due to small sample size.

*OR*, odds ratio; *REF*, reference.

**Table 6:** Regression analysis between primary language and barriers to starting cardiopulmonary resuscitation among survey participants.

	Willingness to start CPR		Fear of doing something wrong		Language barrier	

	OR	95% CI	OR	95% CI	OR	95% CI

Primary Language						
English	REF	REF	REF	REF	REF	REF
Spanish	0.11*	0.05, 0.24*	1.91*	1.02, 3.61*	18.24*	4.63, 101.9*
Age						
13–25 years	0.55	0.19, 1.51	1.3	0.60, 2.80	0.31	0.06, 1.24
26–40 years	REF	REF	REF	REF	REF	REF
41–55 years	1.35	0.58, 3.19	0.49*	0.24, 0.98*	1.14	0.36, 3.57
56–70 years	4.79*	2.07, 11.51*	0.12*	0.04, 0.28*	3.5	0.82, 15.73
71–85 years	1.46	0.55, 3.95	0.39	0.14, 1.02	7.16	0.95, 60.23
>85 years	0.78	0.09, 5.76	0.37	0.02, 2.99	**	**
Education Level						
Primary School	1.48	0.20, 6.95	0.54	0.13, 1.96	1.4	0.25, 6.65
High School	REF	REF	REF	REF	REF	REF
2 years of college	1.09	0.49, 2.41	1.3	0.58, 2.93	0.49	0.08, 2.13
4 years of college	0.83	0.38, 1.82	1.49	0.73, 3.06	0.42	0.10, 1.48
Graduate School	0.8	0.33, 1.92	1.4	0.57, 3.40	0.36	0.01, 3.18
Household Income						
Under $10,000	REF	REF	REF	REF	REF	REF
$10,000–$30,000	2.11	0.71, 6.65	0.67	0.27, 1.63	0.51	0.16, 1.55
$30,000–$60,000	2.23	0.81, 6.52	0.67	0.29, 1.56	0.42	0.14, 1.22
> $60,0000	1.09	0.39, 3.17	1.24	0.52, 2.99	0.07*	0.00, 0.51*

Spanish-speaking respondents were 18.24 times more likely to indicate language as a barrier to starting CPR, compared to English-speaking respondents (95% CI, 4.63–101.98; P < .001). Respondents who had an annual household income over $60,000 were 93% less likely to indicate language as a barrier to starting CPR (OR 0.07; 95% CI, 0.00–0.51; P = .03).

* Statistically significant estimates at P < .05.

** Estimate unable to be obtained due to small sample size.

*OR*, odds ratio; *REF*, reference.

## References

[1] Tsao CW, Aday AW, Almarzooq ZI (2022). Heart Disease and Stroke Statistics—2022 Update: a report from the American Heart Association. Circulation.

[2] Brady WJ, Mattu A, Slovis CM (2019). Lay responder care for an adult with out-of-hospital cardiac arrest. N Engl J Med.

[3] Perkins GD, Callaway CW, Haywood K (2021). Brain injury after cardiac arrest. Lancet.

[4] Dainty KN, Colquitt B, Bhanji F (2022). Understanding the importance of the lay responder experience in out-of-hospital cardiac arrest: a scientific statement from the American Heart Association. Circulation.

[5] Marco CA, Larkin GL (2008). Cardiopulmonary resuscitation: knowledge and opinions among the U.S. general public: state of the science-fiction. Resuscitation.

[6] McNally B, Robb R, Mehta M (2011). Out-of-hospital cardiac arrest surveillance – Cardiac Arrest Registry to Enhance Survival (CARES), United States, October 1, 2005–December 31, 2010. Morb Mortal Wkly Rep Surveill Summ Wash DC 2002.

[7] Valenzuela TD, Spaite DW (2000). Outcomes of rapid defibrillation by security officers after cardiac arrest in casinos. N Engl J Med.

[8] Chen C, Lo CYZ, Ho MJC (2024). Global sex disparities in bystander cardiopulmonary resuscitation after out-of-hospital cardiac arrest: a scoping review. J Am Heart Assoc.

[9] Garcia RA, Spertus JA, Girotra S (2022). Racial and ethnic differences in bystander CPR for witnessed cardiac arrest. N Engl J Med.

[10] Chu K, Swor R, Jackson R (1998). Race and survival after out-of-hospital cardiac arrest in a suburban community. Ann Emerg Med.

[11] Brookoff D, Kellermann AL, Hackman BB (1994). Do Blacks get bystander cardiopulmonary resuscitation as often as Whites?. Ann Emerg Med.

[12] Bradley SM, Fahrenbruch CE, Meischke H (2011). Bystander CPR in out-of-hospital cardiac arrest: the role of limited English proficiency. Resuscitation.

[13] Nuño T, Bobrow BJ, Rogge-Miller KA (2017). Disparities in telephone CPR access and timing during out-of-hospital cardiac arrest. Resuscitation.

[14] Perera N, Birnie T, Ngo H (2021). “I’m sorry, my English not very good”: tracking differences between language-barrier and non-language-barrier emergency ambulance calls for out-of-hospital cardiac arrest. Resuscitation.

[15] Centers for Disease Control and Prevention (2024). Social Determinants of Health (SDOH).

[16] Office of Disease Prevention and Health Promotion (2020). Social Determinants of Health - Healthy People 2030.

[17] Demirovic J (2004). Cardiopulmonary resuscitation programs revisited: results of a community study among older African Americans. Am J Geriatr Cardiol.

[18] Anderson ML, Cox M, Al-Khatib SM (2014). Cardiopulmonary resuscitation training rates in the United States. JAMA Intern Med.

[19] Sasson C, Haukoos JS, Ben-Youssef L (2015). Barriers to calling 911 and learning and performing cardiopulmonary resuscitation for residents of primarily Latino, high-risk neighborhoods in Denver, Colorado. Ann Emerg Med.

[20] Berkowitz D, Cohen JS, McCollum N (2023). Delays in treatment and disposition attributable to undertriage of pediatric emergency medicine patients. Am J Emerg Med.

[21] Humans Rights Watch (2014). US: Immigrants ‘afraid to call 911’.

[22] Harris PA, Taylor R, Minor BL (2019). The REDCap consortium: building an international community of software platform partners. J Biomed Inform.

[23] Harris PA, Taylor R, Thielke R (2009). Research electronic data capture (REDCap)—A metadata-driven methodology and workflow process for providing translational research informatics support. J Biomed Inform.

[24] R Core Team (2023). R: A Language and environment for statistical computing.

[25] Rdocumentation (2022). Tableone Package Version 0.13.2.

[26] Sasson C, Haukoos JS, Bond C (2013). Barriers and facilitators to learning and performing cardiopulmonary resuscitation in neighborhoods with low bystander cardiopulmonary resuscitation prevalence and high rates of cardiac arrest in Columbus, OH. Circ Cardiovasc Qual Outcomes.

[27] US Census Bureau (2023). Custom Table -- Roanoke Valley-Allegheny Regional Commission (Roanoke, Salem Cities & Vinton Town) PUMA; Virginia, American Community Survey 5-Year Estimates Public Use Microdata Sample.

