# Supplementary material for: Impact of Primary Spoken Language as a Social Determinant of Health on Cardiopulmonary Education and Use: Pilot Study
Source: West J Emerg Med. 2026 Jan 3;27(1):1–9. doi: 10.5811/westjem.47910 (PMC12815535; doi:10.5811/westjem.47910)
Supplement: Supplementary file 1 [file wjem-27-1-s001.pdf]

1. What is your age?
   a. 13-25 years old   d. 56 – 70 years old
   b. 26-40 years old   e. 71 – 85 years old
   c. 41-55 years old   f. over 85 years old

2. What is your gender?   a. Male   b. Female   c. Other

3. Which of the following best describes you? (circle multiple if applicable)

Asian or Pacific Islander   Black or African American   Hispanic or Latino   Native American or Alaskan Native   White or Caucasian

Other:

4. Where do you live (ZIP code)? ___________________________________

5. What is your highest level of education completed (circle one)?

   Primary school   High school   2 years of college   4 years of college   Grad school

6. What is your total yearly household income?
   a. under 10,000   b. 10,000-30,000
   c. 30,000-60,000   d. over 60,000

7. Which of the following would prevent you from calling 911? **(circle all that apply)**

   Law Enforcement   Immigration Status   Cost/Money

   Fear of doing something wrong   Language barrier   Concern for violence

   I would have no problem calling 911   Other_______________________

8. Which of these would prevent you from starting CPR? **(circle all that apply)**

Law Enforcement   Immigration Status   Cost/Money

Fear of doing something wrong   Language barrier   Concern for violence

I would have no problem starting CPR   Other_____________________

9. Have you ever received CPR or AED training prior to today?

   Yes, CPR   Yes, AED   Yes, Both   No

10. If no, what has prevented you from receiving this education?

    a. Didn’t think it was important
    b. Didn’t think I was qualified
    c. Didn’t know it existed
    d. Couldn’t fit it in my schedule
    e. Cost
    f. Other _____________________

Por favor llene ambos lados de la página

1. ¿Cuál es tu edad actual?
    a. 13 a 25 años d. 56 a 70 años
    b. 26 a 40 años e. 71 a 85 años
    c. 41 a 55 años f. más de 85 años
2. ¿Cuál es tu género? a. Masculino b. Femenino c. otro
3. ¿Cuál(es) de los siguientes te describe? (marque todas pertinentes)

Asiático o Isleño del Pacífico Negro o Afroamericano. Hispano o Latino
Indígena Americana o Nativo de Alaska Blanco o Caucásico

4. ¿Dónde vives (Código ZIP)?
5. ¿Cuál es su nivel más alto de educación que ha completado?

Primaria Secundaria/colegio 2 años de universidad Universitaria/licenciatura Posgrado

6. ¿Cuál es el ingreso anual combinado de su hogar?
    a. Menos de 10,000
    b. 10,000-30,000
    c. 30,000-60,000
    d. más de 60,000
7. ¿Cuál(es) de las siguientes opciones le impediría llamar al 911 (marque las que correspondan)?
    a. La policía
    b. Costo/Dinero
    c. Estatus migratorio
    d. Miedo de hacer algo mal
    e. La barrera del idioma
    f. Preocupación por la violencia
    g. No tendría problemas llamando 911
    h. Otro _________________
8. ¿Cuál(es) de las siguientes opciones le impediría iniciar RCP (marque las que correspondan)?
    a. La policía
    b. Costo/Dinero
    c. Estado migratorio
    d. Miedo de hacer algo mal
    e. La barrera del idioma

Por favor llene ambos lados de la página

f. Preocupación por la violencia
g. No tendría problemas empezando RCP
h. Otro ________________

9. ¿Ha recibido algún capacitación en RCP o DEA antes?

Sí, RCP   Sí, DEA   Sí, ambos   No

10. Si no, ¿Qué ha prevenido que recibiera esta educación?

a. No pensaba que era importante
b. No pensaba que podía
c. No sabía que existía
d. Falta de tiempo
e. Costo
f. Otro _____________________
