# Supplementary material for: Impact of Primary Spoken Language as a Social Determinant of Health on Cardiopulmonary Education and Use: Pilot Study
Source: West J Emerg Med. 2026 Jan 3;27(1):1–9. doi: 10.5811/westjem.47910 (PMC12815535; doi:10.5811/westjem.47910)
Supplement: Supplementary file 2 [file wjem-27-1-s002.pdf]

<u>**Zip Code**</u>	<u>**City**</u>	<u>**State**</u>	<u>**County**</u>
24011	Roanoke	VA	Roanoke City
24012	Bonsack	VA	Roanoke City
24013	Roanoke	VA	Roanoke City
24014	Garden City	VA	Roanoke City
24015	Grandin Road	VA	Roanoke City
24016	Roanoke	VA	Roanoke City
24017	Melrose	VA	Roanoke City
24018	Cave Spring/Poages Mill/Roanoke	VA	Roanoke
24019	Hollins/Roanoke	VA	Botetourt
24055	Bassett/Bassett Forks	VA	Henry
24060	Blacksburg	VA	Montgomery
24064	Blue Ridge	VA	Botetourt
24066	Buchanan/Lithia	VA	Botetourt
24070	Catawba	VA	Roanoke
24079	Copper Hill	VA	Floyd
24083	Daleville	VA	Botetourt
24087	Elliston/Ironto/Lafayette	VA	Roanoke
24090	Fincastle	VA	Botetourt
24091	Alum Ridge/Floyd	VA	Floyd
24092	Glade Hill/Gladehill	VA	Franklin
24101	Hardy	VA	Bedford
24112	Martinsville	VA	Henry
24121	Moneta/Scruggs	VA	Bedford
24141	Fairlawn	VA	Radford
24151	Franklin Heights/Roucky Mount	VA	Franklin
24153	Bennett Springs/Fort Lewis/Glenvar/Hanging Rock/Kesslers Mill/Mason Cove	VA	Salem
24175	Haymakertown/Troutville	VA	Botetourt
24176	Union Hall	VA	Franklin
24179	Chamlissburg/Stewartsville/Vinton	VA	Roanoke
24184	Burnt Chimneys/Wirtz	VA	Franklin
24551	Forest	VA	Bedford
